# Enhancing prognostic prediction in hepatocellular carcinoma post-TACE: a machine learning approach integrating radiomics and clinical features

**DOI:** 10.3389/fmed.2024.1419058

**Published:** 2024-07-17

**Authors:** Mingqi Zhang, Bingling Kuang, Jingxuan Zhang, Jingyi Peng, Haoming Xia, Xiaobin Feng, Liang Peng

**Affiliations:** ^1^Department of Gastroenterology, First Affiliated Hospital of Guangzhou Medical University, Guangzhou Medical University, Guangzhou, Guangdong, China; ^2^The Second Clinical School of Guangzhou Medical University, Guangzhou Medical University, Guangzhou, Guangdong, China; ^3^Nanshan College, Guangzhou Medical University, Guangzhou, Guangdong, China; ^4^School of Clinical Medicine, Tsinghua University, Beijing, China; ^5^Hepato-Pancreato-Biliary Center, Beijing Tsinghua Changgung Hospital, School of Clinical Medicine, Tsinghua University, Beijing, China

**Keywords:** machine learning, hepatocellular carcinoma, prognosis, radiomics, clinical features

## Abstract

**Objective:**

This study aimed to investigate the use of radiomics features and clinical information by four machine learning algorithms for predicting the prognosis of patients with hepatocellular carcinoma (HCC) who have been treated with transarterial chemoembolization (TACE).

**Methods:**

A total of 105 patients with HCC treated with TACE from 2002 to 2012 were enrolled retrospectively and randomly divided into two cohorts for training (*n* = 74) and validation (*n* = 31) according to a ratio of 7:3. The Spearman rank, random forest, and univariate Cox regression were used to select the optimal radiomics features. Univariate Cox regression was used to select clinical features. Four machine learning algorithms were used to develop the models: random survival forest, eXtreme gradient boosting (XGBoost), gradient boosting, and the Cox proportional hazard regression model. The area under the curve (AUC) and C-index were devoted to assessing the performance of the models in predicting HCC prognosis.

**Results:**

A total of 1,834 radiomics features were extracted from the computed tomography images of each patient. The clinical risk factors for HCC prognosis were age at diagnosis, TNM stage, and metastasis, which were analyzed using univariate Cox regression. In various models, the efficacy of the combined models generally surpassed that of the radiomics and clinical models. Among four machine learning algorithms, XGBoost exhibited the best performance in combined models, achieving an AUC of 0.979 in the training set and 0.750 in the testing set, demonstrating its strong prognostic prediction capability.

**Conclusion:**

The superior performance of the XGBoost-based combined model underscores its potential as a powerful tool for enhancing the precision of prognostic assessments for patients with HCC.

## 1 Introduction

Primary liver cancer is the sixth most common cancer globally and the third leading cause of cancer-related deaths worldwide. Hepatocellular carcinoma (HCC) constitutes approximately 75–85% of all primary liver cancer cases (1). There are many treatment options for HCC, including local ablation therapy, liver transplantation, liver resection, transarterial chemoembolization (TACE), radiation therapy, and systemic treatment. Presently, surgical resection stands as the principal treatment approach for HCC. However, due to HCC often presenting without distinct early symptoms, many patients are already at the intermediate stage [Barcelona Clinic Liver Cancer (BCLC) B stage] according to the BCLC staging system at the time of diagnosis, missing the optimal timing for treatment (2). This limits the choice of treatment options and affects the patient’s prognosis.

The TACE is a minimally invasive technique that uses imaging guidance to diminish the blood supply to a tumor. Through a catheter inserted into an artery, contrast materials are administered to block the tumor’s blood vessels, thereby halting the growth of new blood vessels and causing cell death in the tumor. According to European and American HCC management guidelines, TACE is a commonly used interventional treatment method for patients with intermediate to advanced HCC, effectively delaying disease progression and providing a chance of survival for some patients (3–7).

Owing to considerable variability within the patients, the effectiveness and safety of TACE treatment for individuals with intermediate to advanced HCC can differ (8). Therefore, before treatment begins, an objective method must be available to accurately predict the prognosis of patients with HCC treated with TACE. For patients with HCC who are not expected to benefit from TACE, alternative treatment methods should be considered, such as using sorafenib or lenvatinib, while preserving liver function as much as possible to extend overall survival (OS) (9–11).

In recent years, with the advancement of imaging technology and the development of big data analysis techniques, radiomics has emerged as a new research field. Radiomics transforms medical images into high-dimensional, quantitative, and minable data through deep feature extraction and data analysis, quantifying tumor phenotypic characteristics and heterogeneity, and is considered a potential biomarker for personalized cancer treatment (12). Moreover, machine learning methods excel at handling the intricate interactions among complex variables, which is difficult for traditional models (13). Nowadays, some studies have reported substantial progress in the diagnosis, treatment response, and prognosis prediction of HCC by combining radiomics features, clinical information, and computer technology, especially in TACE treatment for patients with HCC (14–16). However, predicting prognosis for patients with HCC with the use of radiomics or clinical information by different machine learning algorithms has not been fully explored, and their performance may vary in different scenarios.

Therefore, this study aimed to develop and validate different prediction models using four machine learning algorithms. It includes radiomics, clinical, and combined models incorporating clinical information and radiomics features. The purpose of these models is to predict the OS of patients with HCC after TACE treatment, providing new insights and effective strategies for selecting treatment options.

## 2 Materials and methods

### 2.1 Patients

To obtain the requisite data, we used the public data repository, The Cancer Imaging Archive (TCIA, https://www.cancerimagingarchive.net/) database. We collected the data of 105 patients with HCC who were treated with TACE from 2002 to 2012. The inclusion criteria specified that TACE must be the sole first-line or initial bridging therapy, accompanied by the availability of multiphasic contrast material enhanced computed tomography (CT) images at baseline, free from any image artifacts such as surgical clips. More information can be found in previous studies (17–19).

Patients were randomly divided into two categories at a ratio of 7:3: a training cohort (*n* = 74) and a testing cohort (*n* = 31). The training cohort was used to build the predictive models, while the testing cohort was used to validate the performance of the predictive model.

### 2.2 Image acquisition and segmentation

The dataset used was taken from TCIA and consisted of CT images from 105 patients. More information can be found in the TCIA database and previous studies (17–19). For the dataset from TCIA, expert radiologists meticulously annotated CT images from 105 patients using specialized software, focusing on the precise delineation of tumors and anatomical structures. Adhering to a standardized protocol, they outlined regions of interest on each slice, with their work undergoing rigorous review in consensus meetings to ensure accuracy and consistency.

### 2.3 Radiomics features

#### 2.3.1 Radiomics features extraction

Feature extraction was based on Python 3.7 and implemented using the PyRadiomics software^1^ (20). The algorithms for obtaining radiomics features were referenced from the Image Biomarker Standardization Initiative (21). The extracted radiomics features can be divided into three groups: (1) first-order statistical features; (2) shape features, including two-dimensional and three-dimensional characteristics; and (3) texture features, including gray level co-occurrence matrix, gray level run length matrix, gray level size zone matrix, gray level dependence matrix, and neighborhood gray-tone difference matrix.

#### 2.3.2 Radiomics features selection

Initially, within the training dataset, the Spearman rank correlation coefficient was used to determine the inter-feature correlations, retaining one feature from any pair with a correlation coefficient exceeding 0.9 to eliminate highly redundant features. To maximally preserve the descriptive power of features, a greedy recursive elimination strategy was applied for feature filtering, wherein the most redundant feature in the current set was removed at each iteration.

Subsequently, further selection was performed using random forests, an ensemble learning method based on decision trees that assesses the contribution of each feature to the model’s predictive performance. By evaluating the role of feature splits in the trees, random forests determined the extent to which feature splits improve model accuracy. Feature importance helped identify the most influential features for predicting the target variable (survival time).

Finally, univariate Cox proportional regression analysis was used to evaluate the impact of each variable on survival time. In this analysis, each variable was examined in relation to survival time separately, to ascertain its effect on survival risk. This method identified variables for subsequent model construction by calculating the hazard ratio and corresponding statistical significance (*p*-value) for each feature, incorporating variables from univariate regression analyses with a *p*-value of <0.05.

### 2.4 Clinical features selection

In the initial data preparation phase, features with more than 20% missing values were excluded to maintain the integrity and reliability of the dataset. This step was crucial to ensuring the robustness and comprehensiveness of the clinical information used for modeling. Following this, to simplify the model and enhance its interpretability, continuous variables were converted into binary variables (dichotomization). This process involved setting a threshold for each feature, above which values were coded as 1 and below as 0, thus categorizing patients into two distinct groups based on each feature’s presence or absence.

For feature selection, univariate Cox proportional hazards regression analysis was used on the training set. This statistical method was used to assess the impact of each feature on the OS of patients with HCC, identifying variables that significantly affected the outcome. Features with a *p*-value less than 0.1 in this analysis were considered statistically significant and were selected for further modeling.

### 2.5 Construction and validation of models for survival prediction

The application of machine learning algorithms was carefully tailored, with specific parameters set to optimize their performance for radiomics and clinical data. The Cox proportional hazard regression model (Coxph) was parameterized to evaluate the risk factors with adjustments to its baseline hazard function and regression coefficients to suit the survival data. For the random survival forest (RSF) algorithm, many decision trees were constructed to improve prediction accuracy, with parameters such as the number of trees, maximum depth, and minimum samples per leaf tuned to prevent overfitting while capturing the complex interactions within the data. Gradient Boosting was utilized to minimize errors sequentially using decision trees, where the learning rate and the number of trees were critical parameters to balance bias and variance effectively. Finally, eXtreme gradient boosting (XGBoost) was used, as it is known for its efficiency and scalability. Parameters such as the learning rate, maximum depth of trees, and the number of estimators were optimized to enhance the model’s ability to accurately predict the outcomes of patients with HCC. To validate these models, a 5-fold cross-validation method was used, assessing their prediction accuracy through the average area under the curve (AUC) on the testing cohort, thus ensuring a robust evaluation of each algorithm’s predictive power.

### 2.6 Statistical analysis

Statistical analysis was conducted using R software version 4.2.3.^2^ The normality of continuous data was tested using the Shapiro–Wilk test, with normally distributed data presented as mean ± standard deviation (x̅ ± s), and differences between two groups were analyzed using independent sample *t*-tests. Non-normally distributed data were presented as M (Q1, Q3), with differences between groups analyzed using the Mann–Whitney *U*-test. Categorical data were compared using the chi-square test, with a *p*-value of <0.05 considered statistically significant. OS was regarded as the primary outcome. Receiver operating characteristic (ROC) curves were generated using the “pROC” package. The model that achieved the highest AUC was chosen as the best prediction model. ROC curves and C-index were used to assess the predictive capability of different models for the prognosis of patients with HCC treated with TACE.

## 3 Results

### 3.1 Clinical baseline characteristics of patients

Table 1 displays the clinical characteristics of patients in the training cohort (*n* = 74) and the testing cohort (*n* = 31). A total of 12, 24, 66, and 3 patients were in BCLC stages A, B, C, and D, respectively. In the training cohort, most patients (71.62%) were diagnosed at age over 60 years, and 66.22% were men. Additionally, most patients were not diagnosed with vascular invasion (82.43%) or diabetes (66.22%). Moreover, 74.32% of the patients had cirrhosis, and 94.59% had metastasis. No significant differences were observed in clinical characteristics between the training and testing cohorts (*p* > 0.05).

**Table 1:** Baseline of 105 enrolled patients from TCIA.

Characteristics	Training cohort (*N* = 74)	Testing cohort (*N* = 31)	*P*
OS, No. (%)			0.98
Alive	9 (12.16%)	3 (9.68%)	
Dead	65 (87.84%)	28 (90.32%)	
OS.time (weeks), mean (*SD*)	128.03 (106.22)	123.13 (97.57)	0.93
Hepatitis, No. (%)			1.00
No	38 (51.35%)	16 (51.61%)	
Yes	36 (48.65%)	15 (48.39%)	
Age at diagnosis (years), No. (%)			1.00
<=60	21 (28.38%)	9 (29.03%)	
>60	53 (71.62%)	22 (70.97%)	
Sex, No. (%)			0.80
Male	49 (66.22%)	19 (61.29%)	
Female	25 (33.78%)	12 (38.71%)	
Smoking, No. (%)			0.21
No	29 (39.19%)	17 (54.84%)	
Yes	45 (60.81%)	14 (45.16%)	
Alcohol, No. (%)			0.38
No	32 (43.24%)	17 (54.84%)	
Yes	42 (56.76%)	14 (45.16%)	
Diabetes, No. (%)			1.00
No	49 (66.22%)	21 (67.74%)	
Yes	25 (33.78%)	10 (32.26%)	
Cirrhosis, No. (%)			1.00
No	19 (25.68%)	8 (25.81%)	
Yes	55 (74.32%)	23 (74.19%)	
Child-Pugh, No. (%)			0.29
A	63 (85.14%)	23 (74.19%)	
B or C	11 (14.86%)	8 (25.81%)	
Tumor nodularity, No. (%)			0.95
Uninodular	36 (48.65%)	16 (51.61%)	
Multinodular	38 (51.35%)	15 (48.39%)	
Vascular invasion, No. (%)			0.16
No	61 (82.43%)	21 (67.74%)	
Yes	13 (17.57%)	10 (32.26%)	
Metastasis, No. (%)			0.71
No	70 (94.59%)	28 (90.32%)	
Yes	4 (5.41%)	3 (9.68%)	
AFP(ng/mL), No. (%)			0.76
<400	54 (72.97%)	21 (67.74%)	
>=400	20 (27.03%)	10 (32.26%)	
Okuda, No. (%)			0.26
Stage I	53 (71.62%)	18 (58.06%)	
Stage II or III	21 (28.38%)	13 (41.94%)	
TNM, No. (%)			1.00
Stage I or II	40 (54.05%)	17 (54.84%)	
Stage III or IV	34 (45.95%)	14 (45.16%)	
BCLC, No. (%)			0.16
Stage A or B	29 (39.19%)	7 (22.58%)	
Stage C or D	45 (60.81%)	24 (77.42%)	

### 3.2 Radiomics features screening results

**Figure 1:**
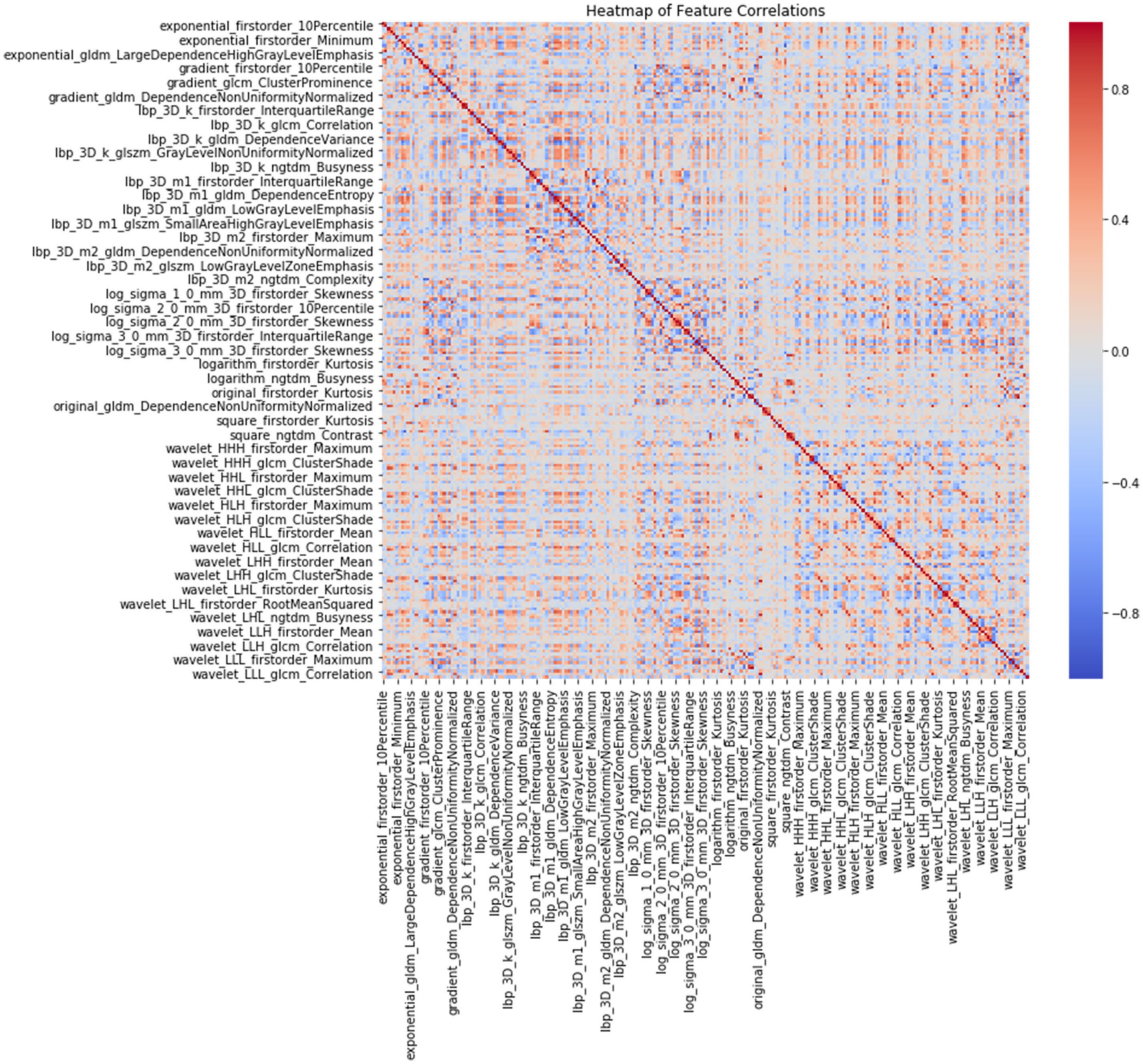
Heatmap of 233 radiomics features correlations according to Spearman rank correlation coefficient.

**Figure 2:**
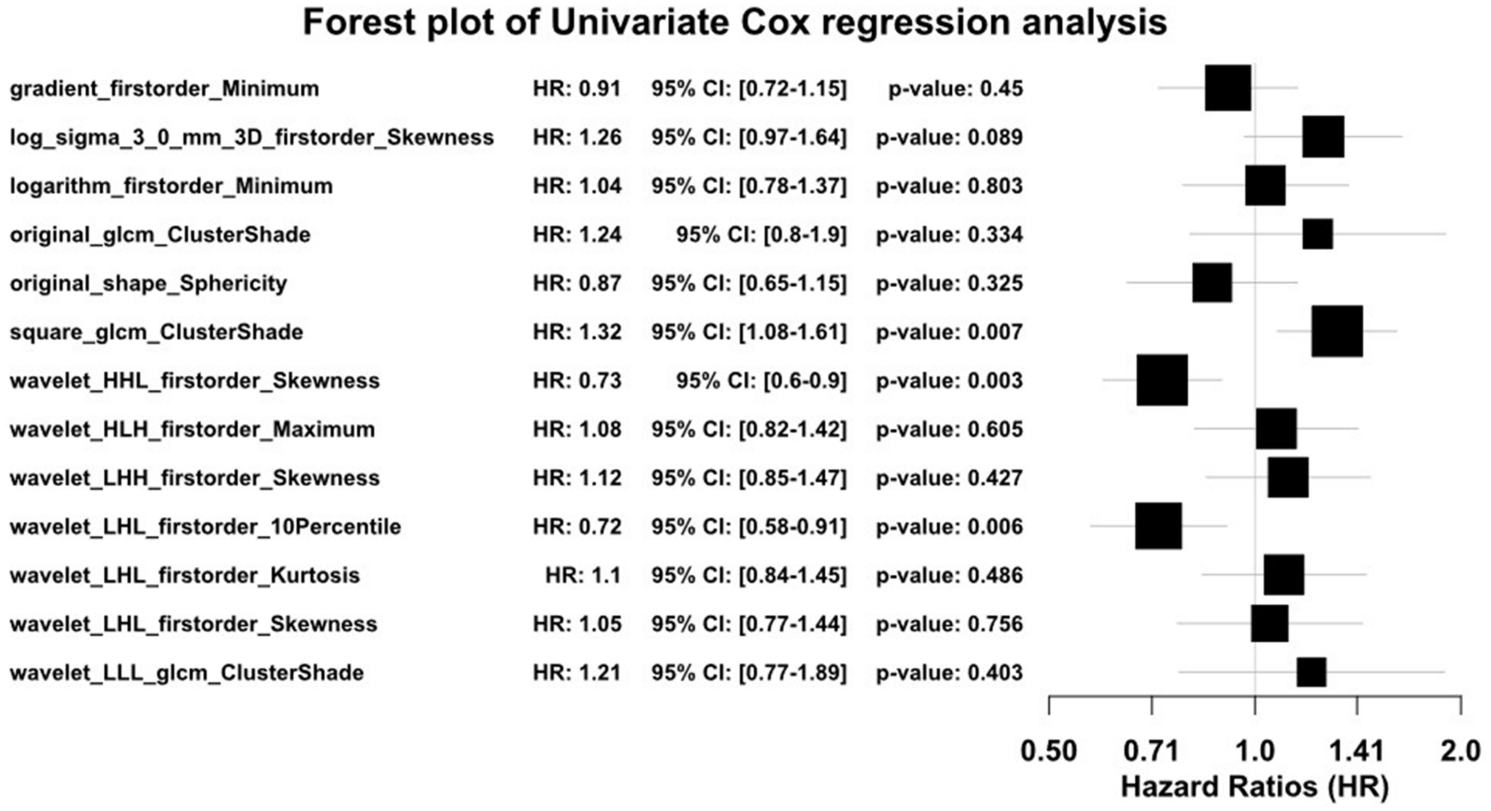
Forest plot of radiomics features selection with univariate Cox regression analysis.

A total of 1,834 radiomics features were initially extracted. Using the Spearman rank correlation coefficient to assess the inter-feature correlations, 233 features were retained. Subsequent selection through random forests resulted in the preservation of 13 features owing to the importance scores. Finally, univariate Cox regression analysis included three variables (*p* < 0.05), comprising two first-order features and one texture feature. Among these, the feature square_glcm_ClusterShade made the most significant contribution. Detailed information about the process is depicted in Figures 1, 2. All selected features were utilized in constructing the radiomics and combined models.

### 3.3 Clinical characteristics included analysis

The clinical characteristics of patients in the training cohort are presented in Table 2. Based on the univariate Cox proportional hazards regression analysis within the training cohort (*p* ≤ 0.1), only age at diagnosis, TNM staging, and metastasis were associated with OS in patients with HCC. Age at diagnosis helped in understanding the survival prognosis, with older age potentially indicating a poorer outcome. The TNM stage, an indicator of cancer progression, provided crucial information on tumor size, lymph node involvement, and the extent of metastasis. The presence of metastasis, indicating the spread of cancer to other parts of the body, was another critical factor influencing survival rates. These findings underscored the importance of these variables in predicting the survival outcomes of patients with HCC. They were used to refine the predictive model for better accuracy and clinical relevance.

**Table 2:** Clinical variables for predicting survival in the univariate Cox analysis.

Variable	Hazard ratio	HR_CI_Lower	HR_CI_Upper	*P*
Progressed	1.43	0.87	2.36	0.16
Hepatitis	0.72	0.44	1.19	0.20
Age at diagnosis	1.79	0.99	3.23	0.05
Sex	1.46	0.87	2.45	0.16
Smoking	1.08	0.64	1.82	0.76
Alcohol	0.85	0.52	1.41	0.54
Family history of cancer	1.05	0.63	1.74	0.86
Family history of liver cancer	0.82	0.30	2.28	0.71
Diabetes	1.16	0.69	1.94	0.57
Personal history of cancer	1.07	0.54	2.11	0.84
Cirrhosis	0.69	0.40	1.20	0.19
Performance status	1.23	0.75	2.02	0.40
Child-Pugh	1.42	0.72	2.80	0.31
Tumor nodularity	1.24	0.76	2.04	0.39
Vascular invasion	1.49	0.81	2.75	0.20
Metastasis	6.53	2.22	19.25	0.00
Lymph nodes	1.23	0.61	2.49	0.57
Portal Vein Thrombosis	1.76	0.86	3.57	0.12
Tumor involvement	0.91	0.48	1.71	0.77
AFP	1.15	0.65	2.04	0.64
CLIP	1.49	0.72	3.05	0.28
Okuda	1.02	0.60	1.75	0.93
TNM	1.53	0.93	2.52	0.09
BCLC	1.21	0.73	2.00	0.47

### 3.4 Model performance

**Figure 3:**
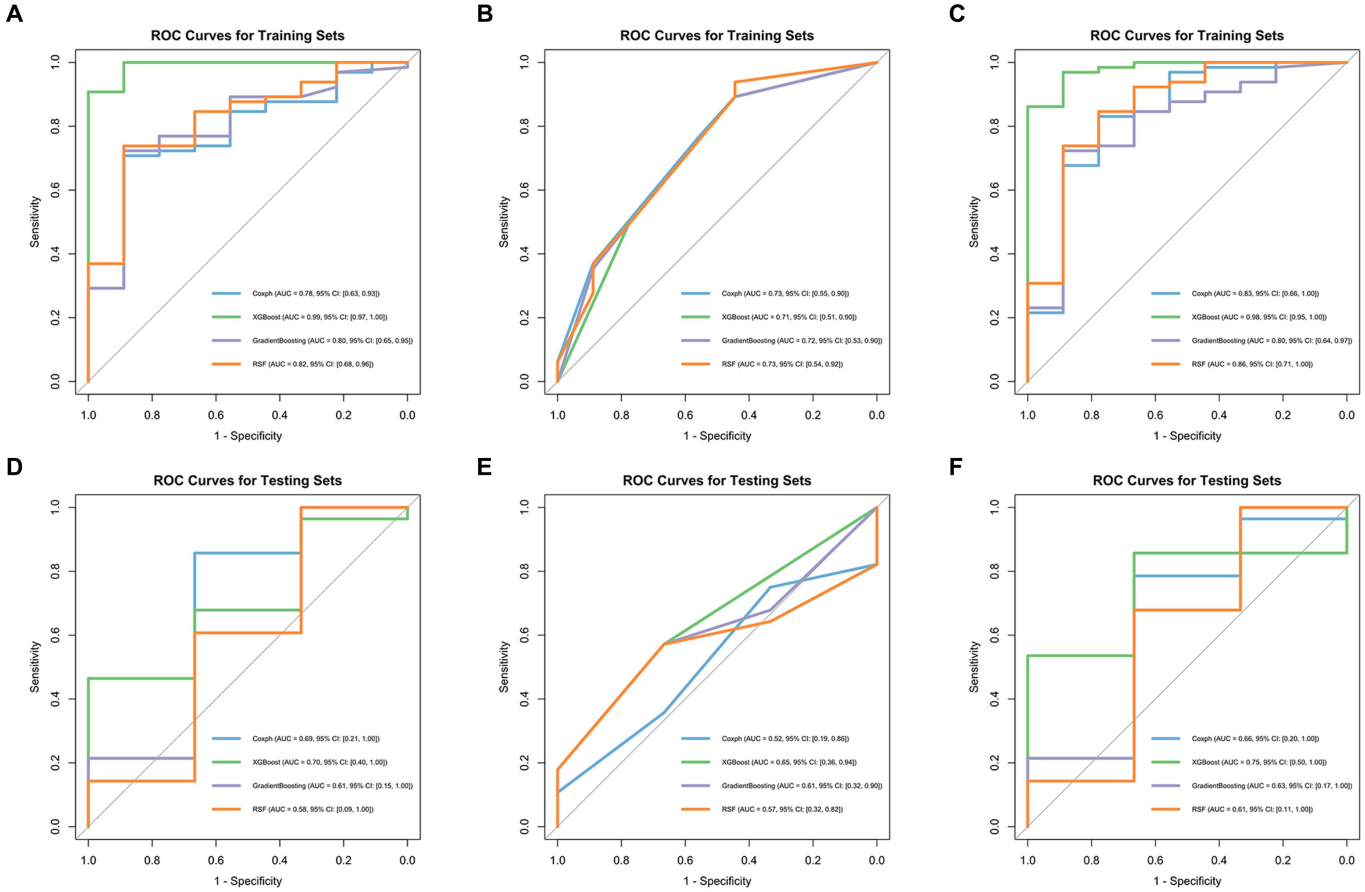
ROC curves of four machine learning algorithms in the training cohort: radiomics models **(A)**, clinical models **(B)**, and combined models **(C)**, in the testing cohort: radiomics models **(D)**, clinical models **(E)**, and combined models **(F)**.

Model performance is shown in Tables 3, 4. ROC curves for each model in the training and testing cohorts are shown in Figure 3. Among all machine learning models, the evaluation of combined models generally outperformed the radiomics or clinical models in predicting HCC prognosis in the training and testing cohorts. Specifically, the XGBoost model in the combined models showed the best performance, achieving an AUC of 0.979 in the training cohort and an AUC of 0.750 in the testing cohort, demonstrating strong prognostic prediction capability. The AUC of the combined model was significantly higher than that of the XGBoost clinical model (AUC = 0.706) and the XGBoost radiomics model (AUC = 0.990). In contrast, the clinical model exhibited a lower AUC, with particularly weaker performance observed in the Coxph and RSF algorithms. In the training cohort, the Coxph and RSF algorithms demonstrated an AUC of 0.726 and 0.728, respectively, while in the testing cohort, the AUC decreased to 0.524 and 0.571, respectively. The clinical models displayed consistent accuracy across all algorithms, with an accuracy of 0.838 in the training and 0.742 in the testing cohorts. Conversely, the radiomics models and the combined models exhibited significant fluctuations in accuracy across different algorithms.

**Table 3:** Performance of different machine learning algorithms in the training cohort.

	AUC	C-Index	Accuracy	Sensitivity	Precision	F1
Clinical models						
Coxph	0.726	0.604	0.838	0.892	0.892	0.892
Gradient boosting	0.718	0.570	0.838	0.892	0.892	0.892
RSF	0.728	0.579	0.838	0.892	0.892	0.892
XGBoost	0.706	0.556	0.838	0.892	0.892	0.892
Radiomics models						
Coxph	0.781	0.663	0.730	0.708	0.708	0.708
Gradient boosting	0.800	0.652	0.743	0.723	0.723	0.723
RSF	0.822	0.674	0.757	0.738	0.738	0.738
XGBoost	0.990	0.979	0.881	0.892	0.892	0.092
Combined models						
Coxph	0.834	0.692	0.824	0.831	0.831	0.831
Gradient boosting	0.805	0.656	0.743	0.723	0.723	0.723
RSF	0.862	0.696	0.757	0.738	0.738	0.738
XGBoost	0.979	0.985	0.922	0.938	0.938	0.938

**Table 4:** Performance of different machine learning algorithms in the testing cohort.

	AUC	C-Index	Accuracy	Sensitivity	Precision	F1
Clinical models						
Coxph	0.524	0.575	0.742	0.821	0.821	0.821
Gradient boosting	0.613	0.579	0.742	0.821	0.821	0.821
RSF	0.571	0.590	0.742	0.821	0.821	0.821
XGBoost	0.649	0.585	0.742	0.821	0.821	0.821
Radiomics models						
Coxph	0.690	0.615	0.516	0.500	0.500	0.500
Gradient boosting	0.607	0.558	0.613	0.643	0.643	0.643
RSF	0.583	0.554	0.645	0.679	0.679	0.679
XGBoost	0.702	0.534	0.623	0.650	0.650	0.650
Combined models						
Coxph	0.655	0.615	0.806	0.857	0.857	0.857
Gradient boosting	0.631	0.560	0.613	0.607	0.607	0.607
RSF	0.607	0.578	0.645	0.679	0.679	0.679
XGBoost	0.750	0.512	0.758	0.879	0.879	0.879

## 4 Discussion

Primary liver cancer is the sixth most common cancer globally. Most patients are diagnosed at an intermediate stage according to the BCLC staging system (stage B), for which TACE is considered the preferred treatment option. This plays an important role in managing patients with HCC (1, 7). However, the treatment response to TACE among patients with HCC often exhibits considerable individual variability, and liver function usually declines in patients with intermediate-stage HCC compared with healthy individuals. Moreover, TACE is highly likely to impose an additional burden on the liver; therefore, accurate preoperative prediction is crucial for treating and managing patients with HCC (22). In this study, we constructed four machine learning models based on patients’ CT images and clinical information. The combined models outperformed single-feature models in predicting the prognosis of patients with HCC treated with TACE, with the XGBoost combined model demonstrating the best performance.

Recently, the rapid advancement of radiomics has enhanced the accuracy of clinical diagnosis and prognosis assessment. Radiomics extracts tissue and lesion characteristics, converting potential pathological and physiological information in images into mineable high-dimensional quantitative image features for analysis, training, and validation, providing a powerful tool for modern medicine to address clinical problems (23, 24). This applies to HCC as well, where radiomics is extensively used. Liu et al. (25) investigated the OS of patients with HCC after hepatectomy. Feng et al. (26) built a radiomics model to predict the macrotrabecular-massive subtype in patients with HCC. Xia et al. (27) extracted radiomics features from images to predict microvascular invasion status in patients with HCC. Tong et al. (28) and Khodabakhshi et al. (29) have also confirmed that radiomics features can predict the prognosis of many cancers. This suggests the potential of radiomics in predicting the prognosis of patients with HCC after TACE. Therefore, in our study, we opted to incorporate radiomics features into constructing machine learning models. We selected three radiomics features, including two first-order features and one texture feature: square_glcm_ClusterShade, wavelet_HHL_firstorder_Skewness, and wavelet_LHL_firstorder_10Percentile.

However, interpreting the relationship between radiomics features and complex tumor biological processes remains challenging, prompting the inclusion of clinical information in our analysis. Regarding clinical information, our study identified age at diagnosis, metastasis, and TNM stage as significant variables affecting the prognosis of patients with HCC, with metastasis occurrence being the most critical variable based on importance scores. Combined models using clinical information and radiomics features outperformed radiomics or clinical models, echoing findings from previous studies. Ning et al. (30) conducted a study that combined radiomics signatures and clinical information to predict early recurrence in HCC. The combined model demonstrated the highest predictive power in the training and validation datasets, with AUCs of 0.846 and 0.737, respectively. Fang et al. (31) and Geng et al. (32) also drew a similar conclusion consistent with our results, where the combined model was a better predictor than the clinical or radiomics models.

Machine learning is widely applied in the medical field because of its high predictive accuracy (33). To date, various machine learning algorithms have been utilized to predict survival, prognosis, and treatment efficacy in patients with intermediate or advanced HCC treated with TACE. However, previous studies usually limit themselves to a single machine learning algorithm for analysis and modeling (34, 35). Considering the different features and scopes of application of various machine learning algorithms, this study used four advanced algorithms: XGBoost, RSF, gradient boosting, and Coxph to build models predicting the OS of patients with HCC under TACE treatment. Our results suggested that XGBoost performed the best among all machine learning algorithms, especially in the combined model constructed after merging radiomics and clinical features, with an AUC of 0.979 in the training and 0.750 in the testing sets, significantly outperforming other algorithms, thereby proving its strong predictive efficiency. According to other studies, XGBoost has been proven to have a better predictive performance than other machine learning algorithms, consistent with our results (36–38). XGBoost is a scalable and highly accurate machine learning library that breaks through the computational limits of gradient boosting tree algorithms. The XGBoost algorithm iteratively optimizes the structure of trees to minimize the loss function, introduces L1 regularization to reduce the number of leaf nodes of decision trees, and introduces L2 regularization to reduce the weight of leaf nodes of decision trees, among other iterative optimizations, enhancing the model’s generalization ability. XGBoost is distinct from other machine learning algorithms because it captures complex and non-linear relationships between features and outcomes. It efficiently processes complex, high-dimensional data, handles missing values effectively, and prevents overfitting. This makes XGBoost particularly well-suited for high-dimensional data scenarios such as radiomics. Our study demonstrated the great potential of the XGBoost in accurately predicting the prognosis of patients with HCC treated with TACE, especially when based on radiomics features and clinical characteristics. Apart from machine learning, we have also identified other advanced algorithms, such as deep learning, which have achieved notable successes in various fields (39, 40). However, because of the limited sample size in this study, machine learning might be a more suitable choice. In the future, we will obtain more samples for further in-depth research.

The limitations of this study included: (1) The ideal TACE candidates are patients in BCLC stage B; however, most patients (*n* = 70) were in BCLC stages C and D. (2) Data were selected in a single center, and external validation from other research centers is needed to improve the universality of the predictive model. (3) The sample size was small. (4) The relatively small number of patients included could have led to model overfitting, and increasing the number of cases would enhance the model’s generalizability. (5) This study was retrospective, lacking a prospective study, and subject to selection bias. (6) An easy-to-use application designed for machine learning algorithms is lacking.

## 5 Conclusion

The purpose of this study was to investigate how four machine learning algorithms utilize radiomics features and clinical information to predict the prognosis of patients with HCC treated with TACE. By applying feature selection methods and testing various machine learning algorithms, it was found that the combined model notably outperformed those based solely on radiomics or clinical features. Among the four algorithms, XGBoost emerged as the most effective, demonstrating the model’s enhanced predictive power in forecasting patient outcomes. This underscores the potential of integrating radiomics and clinical data through advanced machine learning techniques such as XGBoost to improve prognostic predictions in patients with HCC.

## Data availability statement

Publicly available datasets were analyzed in this study. This data can be found here: https://www.cancerimagingarchive.net/collection/hcc-tace-seg/#citations.

## Author contributions

MZ: Conceptualization, Data curation, Methodology, Software, Writing – original draft. BK: Investigation, Methodology, Writing – original draft. JZ: Data curation, Methodology, Writing – original draft. JP: Software, Writing – original draft. XH: Methodology, Software, Writing – original draft. LP: Conceptualization, Funding acquisition, Writing – review & editing. XF: Writing – original draft, Funding acquisition, Software.

## Footnotes

^1^ http://PyRadiomics.readthedocs.io/en/latest

^2^ http://www.r-project.org

## References

[1] Sung H Ferlay J Siegel RL Laversanne M Soerjomataram I Jemal A. Global Cancer statistics 2020: Globocan estimates of incidence and mortality worldwide for 36 cancers in 185 countries. CA Cancer J Clin. (2021) 71:209–49. doi: 10.3322/caac.21660 33538338

[2] Chakraborty E Sarkar D. Emerging therapies for hepatocellular carcinoma (Hcc). Cancers. (2022) 14:2798. doi: 10.3390/cancers14112798, PMID: 35681776 PMC9179883

[3] Marrero JA Kulik LM Sirlin CB Zhu AX Finn RS Abecassis MM. Diagnosis, staging, and Management of Hepatocellular Carcinoma: 2018 practice guidance by the American Association for the Study of Liver Diseases. Hepatology. (2018) 68:723–50. doi: 10.1002/hep.29913, PMID: 29624699

[4] Heimbach JK Kulik LM Finn RS Sirlin CB Abecassis MM Roberts LR. Aasld guidelines for the treatment of hepatocellular carcinoma. Hepatology. (2018) 67:358–80. doi: 10.1002/hep.29086 28130846

[5] Galle PR Forner A Llovet JM Mazzaferro V Piscaglia F Raoul J-L. Easl clinical practice guidelines: management of hepatocellular carcinoma. J Hepatol. (2018) 69:182–236. doi: 10.1016/j.jhep.2018.03.019, PMID: 29628281

[6] Vogel A Martinelli E Vogel A Cervantes A Chau I Daniele B. Updated treatment recommendations for hepatocellular carcinoma (Hcc) from the Esmo clinical practice guidelines. Ann Oncol. (2021) 32:801–5. doi: 10.1016/j.annonc.2021.02.014, PMID: 33716105

[7] Reig M Forner A Rimola J Ferrer-Fàbrega J Burrel M Garcia-Criado Á. Bclc strategy for prognosis prediction and treatment recommendation: the 2022 update. J Hepatol. (2022) 76:681–93. doi: 10.1016/j.jhep.2021.11.018, PMID: 34801630 PMC8866082

[8] Zq W Yw Z. Transcatheter arterial chemoembolization followed by surgical resection for hepatocellular carcinoma: a focus on its controversies and screening of patients Most likely to benefit. Chin Med J. (2021) 134:2275–86. doi: 10.1097/CM9.0000000000001767, PMID: 34593696 PMC8509936

[9] Kudo M. A new treatment option for intermediate-stage hepatocellular carcinoma with high tumor burden: initial Lenvatinib therapy with subsequent selective Tace. Liver Cancer. (2019) 8:299–311. doi: 10.1159/000502905 31768341 PMC6872999

[10] Arizumi T Ueshima K Minami T Kono M Chishina H Takita M. Effectiveness of Sorafenib in patients with Transcatheter arterial chemoembolization (Tace) refractory and intermediate-stage hepatocellular carcinoma. Liver Cancer. (2015) 4:253–62. doi: 10.1159/000367743, PMID: 26734579 PMC4698649

[11] Kudo M Han K-H Ye S-L Zhou J Huang Y-H Lin S-M. A changing paradigm for the treatment of intermediate-stage hepatocellular carcinoma: Asia-Pacific primary liver Cancer expert consensus statements. Liver Cancer. (2020) 9:245–60. doi: 10.1159/000507370, PMID: 32647629 PMC7325125

[12] Lambin P Leijenaar RTH Deist TM Peerlings J de Jong EEC van Timmeren J. Radiomics: the bridge between medical imaging and personalized medicine. Nat Rev Clin Oncol. (2017) 14:749–62. doi: 10.1038/nrclinonc.2017.141 28975929

[13] Avanzo M Wei L Stancanello J Vallières M Rao A Morin O. Machine and deep learning methods for Radiomics. Med Phys. (2020) 47:e185–202. doi: 10.1002/mp.13678, PMID: 32418336 PMC8965689

[14] Sun Z Shi Z Xin Y Zhao S Jiang H Li J. Contrast-enhanced Ct imaging features combined with clinical factors to predict the efficacy and prognosis for Transarterial chemoembolization of hepatocellular carcinoma. Acad Radiol. (2023) 30:S81–91. doi: 10.1016/j.acra.2022.12.031, PMID: 36803649

[15] Niu XK He XF. Development of a computed tomography-based Radiomics nomogram for prediction of Transarterial chemoembolization refractoriness in hepatocellular carcinoma. World J Gastroenterol. (2021) 27:189–207. doi: 10.3748/wjg.v27.i2.189, PMID: 33510559 PMC7807298

[16] Kong C Zhao Z Chen W Lv X Shu G Ye M. Prediction of tumor response via a pretreatment Mri Radiomics-based nomogram in Hcc treated with Tace. Eur Radiol. (2021) 31:7500–11. doi: 10.1007/s00330-021-07910-0, PMID: 33860832 PMC8452577

[17] Clark K Vendt B Smith K Freymann J Kirby J Koppel P. The Cancer imaging archive (Tcia): maintaining and operating a public information repository. J Digit Imaging. (2013) 26:1045–57. doi: 10.1007/s10278-013-9622-7, PMID: 23884657 PMC3824915

[18] Morshid A Elsayes KM Khalaf AM Elmohr MM Yu J Kaseb AO. A machine learning model to predict hepatocellular carcinoma response to Transcatheter arterial chemoembolization. Radiol Artif Intell. (2019) 1:e180021. doi: 10.1148/ryai.2019180021, PMID: 31858078 PMC6920060

[19] Moawad AW Morshid A Khalaf AM Elmohr MM Hazle JD Fuentes D. Multimodality annotated hepatocellular carcinoma data set including pre-and post-Tace with imaging segmentation. Sci Data. (2023) 10:33. doi: 10.1038/s41597-023-01928-3, PMID: 36653372 PMC9849450

[20] van Griethuysen JJM Fedorov A Parmar C. Computational Radiomics System to Decode the Radiographic Phenotype. Cancer Res. (2017) 77:e104–e107. doi: 10.1158/0008-5472.CAN-17-0339 29092951 PMC5672828

[21] Zwanenburg A Vallières M Abdalah MA. The Image Biomarker Standardization Initiative: Standardized Quantitative Radiomics for High-Throughput Image-based Phenotyping. Radiology. (2020) 295:328–338. doi: 10.1148/radiol.2020191145 32154773 PMC7193906

[22] Raoul J-L Forner A Bolondi L Cheung TT Kloeckner R de Baere T. Updated use of Tace for hepatocellular carcinoma treatment: how and when to use it based on clinical evidence. Cancer Treat Rev. (2019) 72:28–36. doi: 10.1016/j.ctrv.2018.11.002, PMID: 30447470

[23] Lambin P Rios-Velazquez E Leijenaar R Carvalho S van Stiphout RGPM Granton P. Radiomics: extracting more information from medical images using advanced feature analysis. Eur J Cancer. (2012) 48:441–6. doi: 10.1016/j.ejca.2011.11.036 22257792 PMC4533986

[24] Bera K Braman N Gupta A Velcheti V Madabhushi A. Predicting Cancer outcomes with Radiomics and artificial intelligence in radiology. Nat Rev Clin Oncol. (2022) 19:132–46. doi: 10.1038/s41571-021-00560-7, PMID: 34663898 PMC9034765

[25] Liu Y Wei X Zhang X Pang C Xia M Du Y. Ct Radiomics combined with clinical variables for predicting the overall survival of hepatocellular carcinoma patients after hepatectomy. Transl Oncol. (2022) 26:101536. doi: 10.1016/j.tranon.2022.101536, PMID: 36115077 PMC9483805

[26] Feng Z Li H Liu Q Duan J Zhou W Yu X. Ct Radiomics to predict macrotrabecular-massive subtype and immune status in hepatocellular carcinoma. Radiology. (2023) 307:e221291. doi: 10.1148/radiol.221291, PMID: 36511807

[27] Xia T-Y Zhou Z-H Meng X-P Zha J-H Yu Q Wang W-L. Predicting microvascular invasion in hepatocellular carcinoma using Ct-based Radiomics model. Radiology. (2023) 307:e222729. doi: 10.1148/radiol.222729, PMID: 37097141

[28] Tong H Sun J Fang J Zhang M Liu H Xia R. A machine learning model based on pet/Ct Radiomics and clinical characteristics predicts tumor immune profiles in non-small cell lung Cancer: a retrospective multicohort study. Front Immunol. (2022) 13:859323. doi: 10.3389/fimmu.2022.859323, PMID: 35572597 PMC9105942

[29] Khodabakhshi Z Amini M Mostafaei S Haddadi Avval A Nazari M Oveisi M. Overall survival prediction in renal cell carcinoma patients using computed tomography Radiomic and clinical information. J Digit Imaging. (2021) 34:1086–98. doi: 10.1007/s10278-021-00500-y, PMID: 34382117 PMC8554934

[30] Ning P Gao F Hai J Wu M Chen J Zhu S. Application of Ct Radiomics in prediction of early recurrence in hepatocellular carcinoma. Abdom Radiol. (2020) 45:64–72. doi: 10.1007/s00261-019-02198-7, PMID: 31486869

[31] Fang C An X Li K Zhang J Shang H Jiao T. A nomogram based on Ct Radiomics and clinical risk factors for prediction of prognosis of hypertensive intracerebral hemorrhage. Comput Intell Neurosci. (2022) 2022:9751988. doi: 10.1155/2022/9751988, PMID: 36531926 PMC9750770

[32] Geng X Zhang Y Li Y Cai Y Liu J Geng T. Radiomics-clinical nomogram for preoperative lymph node metastasis prediction in esophageal carcinoma. J Radiol. (2024) 97:652–9. doi: 10.1093/bjr/tqae009, PMID: 38268475 PMC11027331

[33] Deo RC. Machine learning in medicine. Circulation. (2015) 132:1920–30. doi: 10.1161/CIRCULATIONAHA.115.001593, PMID: 26572668 PMC5831252

[34] Zhang X He Z Zhang Y Kong J. Prediction of initial objective response to drug-eluting beads Transcatheter arterial chemoembolization for hepatocellular carcinoma using Ct Radiomics-based machine learning model. Front Pharmacol. (2024) 15:1315732. doi: 10.3389/fphar.2024.1315732, PMID: 38344175 PMC10854007

[35] Zhou W Lv Y Hu X Luo Y Li J Zhu H. Study on the changes of Ct texture parameters before and after Hcc treatment in the efficacy evaluation and survival predication of patients with Hcc. Front Oncol. (2022) 12:957737. doi: 10.3389/fonc.2022.957737, PMID: 36387217 PMC9650244

[36] Zhang Y-B Yang G Bu Y Lei P Zhang W Zhang D-Y. Development of a machine learning-based model for predicting risk of early postoperative recurrence of hepatocellular carcinoma. World J Gastroenterol. (2023) 29:5804–17. doi: 10.3748/wjg.v29.i43.5804, PMID: 38074914 PMC10701309

[37] Huang Y Chen H Zeng Y Liu Z Ma H Liu J. Development and validation of a machine learning prognostic model for hepatocellular carcinoma recurrence after surgical resection. Front Oncol. (2021) 10:593741. doi: 10.3389/fonc.2020.593741 33598425 PMC7882739

[38] Peng H-Y Duan S-J Pan L Wang M-Y Chen J-L Wang Y-C. Development and validation of machine learning models for non-alcoholic fatty liver disease. Hepatob Pancreat Dis. (2023) 22:615–21. doi: 10.1016/j.hbpd.2023.03.009, PMID: 37005147

[39] Lilhore UK Dalal S Simaiya S. A cognitive security framework for detecting intrusions in Iot and 5g utilizing deep learning. Comput Secur. (2024) 136:103560. doi: 10.1016/j.cose.2023.103560

[40] Lilhore UK Manoharan P Simaiya S Alroobaea R Alsafyani M Baqasah AM. Hidm: hybrid intrusion detection model for industry 4.0 networks using an optimized Cnn-Lstm with transfer learning. Sensors. (2023) 23:7856. doi: 10.3390/s23187856, PMID: 37765912 PMC10535139

